# Efficacy of antioxidants as a therapy for Alzheimer's disease: a meta-analysis

**DOI:** 10.3389/fnut.2026.1878597

**Published:** 2026-06-23

**Authors:** Jianwei Li, Binghui Chen, Jiajun Jiang, Yingqi Feng, Runze Zhang, Dan Li, Rutong Wang, Zifan Long, Linfeng Ge, Yujia Guo, Ziyu Zhou, Wenhui Zou, Chi Zhong, Liang Liao

**Affiliations:** 1 Department of General Practice, Central Hospital of Xiangtan (The Affiliated Hospital of Hunan University), Yuhu District, Xiangtan, Hunan, China; 2 Clinical Anatomy and Reproductive Medicine Application Institute, Hengyang Medical School, University of South China, Hengyang, Hunan, China

**Keywords:** Alzheimer's disease, antioxidants, cognitive function, neuropsychiatric symptoms, oxidative stress, randomized controlled trial

## Abstract

**Background:**

Oxidative stress plays a central role in the pathogenesis of Alzheimer's disease (AD), contributing to neuronal damage, amyloid-beta aggregation, tau hyperphosphorylation, and neuroinflammation. Although antioxidants have been proposed as potential therapeutic agents, clinical trials have yielded inconsistent results.

**Objective:**

To systematically evaluate the efficacy of various antioxidants—including vitamins, polyphenols, and other antioxidant preparations—on cognitive function, oxidative stress biomarkers, neuropsychiatric symptoms, and disease progression in patients with AD.

**Methods:**

We conducted a systematic search of PubMed, Embase, and Web of Science from inception to October 2, 2025, for randomized controlled trials (RCTs) evaluating antioxidant interventions in AD patients. Study selection, data extraction, and quality assessment were performed independently by multiple reviewers. Meta-analyses were conducted using random-effects models where significant heterogeneity was present (I^2^ > 50%). Outcomes included cognitive function scales (e.g., ADAS-Cog, MMSE), oxidative stress markers, neuropsychiatric symptoms, daily living abilities, neuroimaging measures, and fluid biomarkers.

**Results:**

A total of 23 RCTs comprising 10,537 participants were included. Antioxidants showed mixed effects across outcomes. While certain oxidative stress markers (urinary 8-iso-prostaglandin F2α: SMD 0.75, 95% CI 0.12–1.38) and neuropsychiatric symptoms (NPI: SMD −0.85, 95% CI −1.13 to −0.57) improved significantly, core cognitive endpoints such as ADAS-Cog (SMD 0.01, 95% CI −0.21 to 0.23) and MMSE (SMD 0.19, 95% CI −0.17 to 0.56) showed no significant benefit. Fluid biomarkers including Aβ42, p-tau, and t-tau remained unchanged. High heterogeneity was observed across multiple outcomes, reflecting variability in antioxidant types, dosages, and patient populations.

**Conclusion:**

Antioxidants may improve certain oxidative stress markers and neuropsychiatric symptoms in AD patients but do not consistently enhance core cognitive function or alter AD-specific pathology. Current evidence does not support antioxidants as disease-modifying therapies, though they may serve as adjunctive interventions to improve quality of life and behavioral symptoms. Well-designed RCTs with longer follow-up and standardized protocols are warranted.

**Systematic review registration:**

https://www.crd.york.ac.uk/prospero/display_record.php?ID=CRD420261321660, identifier: CRD420261321660.

## 1 Introduction

Alzheimer's disease (AD) is one of the most prevalent types of senile dementia worldwide, characterized by an irreversible disease course that ultimately leads to death in most patients (1). According to the 2025 Alzheimer's Disease Facts and Figures released by Alzheimer's Association, an estimated 7.2 million Americans aged 65 and older are living with Alzheimer's disease dementia (AD), accounting for 11% of this age group in the United States (2). Defined as a chronic, progressive neurodegenerative disorder, AD is pathologically hallmarked by the accumulation of extracellular β-amyloid (Aβ) plaques and intracellular neurofibrillary tangles (NFTs) composed of hyperphosphorylated tau protein (3–5). Clinically, the disease typically presents with insidious onset of short-term memory impairment, progressing to global cognitive decline—including aphasia, deficits in executive function and visuospatial orientation—and ultimately leading to loss of functional independence and neuropsychiatric symptoms (6–8). Beyond clinical manifestations, the underlying pathogenesis is multifactorial, with the “amyloid cascade hypothesis” standing as the primary pathogenic mechanism—wherein Aβ aggregation triggers tau hyperphosphorylation, synaptic dysfunction, and chronic neuroinflammation, and oxidative stress emerges as a key downstream mediator that amplifies neuronal damage, particularly in the hippocampus and cerebral cortex (9–11). Notably, oxidative stress acts as a core pathological driver throughout the disease course: it not only accelerates the two classic pathological alterations (Aβ deposition and tau hyperphosphorylation) but also directly impairs neuronal and synaptic function, exacerbating cognitive decline. Furthermore, oxidative stress forms a vicious cycle with neuroinflammation and mitochondrial dysfunction, functioning as a central link in the AD pathogenic network (12). Therapeutically, current strategies primarily rely on cholinesterase inhibitors and NMDA receptor antagonists for symptomatic relief, while emerging disease-modifying immunotherapies aim to clear amyloid plaques to slow clinical progression—yet no treatments can reverse or halt disease progression fundamentally (13–16). A growing body of literature has highlighted the potential therapeutic value of antioxidants in AD management, including vitamin-based antioxidants (e.g., vitamin E, vitamin C), natural polyphenols (e.g., curcumin, resveratrol, and tea polyphenols), and other antioxidant agents (e.g., glutathione precursor N-acetylcysteine [NAC], coenzyme Q10, edaravone, mitochondria-targeted antioxidants such as MitoQ) (17–25). However, inconsistent findings across existing clinical trials—attributed to variations in antioxidant types, dosing regimens, and patient populations—highlight the need for a systematic meta-analysis to clarify their efficacy. Therefore, the present meta-analysis aims to systematically evaluate the efficacy of antioxidants (e.g., vitamin E, coenzyme Q10, and polyphenols) in improving cognitive function and slowing disease progression in AD patients, providing evidence-based insights for clinical practice.

## 2 Materials and methods

This study adheres to the Preferred Reporting Items for Systematic Reviews and Meta-Analyses (PRISMA) statement (26). The protocol for this systematic review and meta-analysis has been prospectively registered in the PROSPERO database (registration number: CRD420261321660).

### 2.1 Eligibility criteria

This review includes published Clinical Study and randomized controlled trials (RCTs) evaluating the efficacy of antioxidants—such as vitamins (vitamin E, vitamin C), natural polyphenols (curcumin, resveratrol), and other antioxidant preparations (Alpha-lipoic acid, coenzyme Q10, Omega-3 fatty acids, and Melatonin)—compared to placebo or alternative treatments in patients diagnosed with Alzheimer's disease (AD) using the internationally recognized criteria (e.g., NIA-AA 2021, DSM-5).

### 2.2 Search strategy

We conducted a comprehensive literature search across PubMed, Web of Science, Embase up to October 2, 2025, identifying published Clinical Study and randomized controlled trials (RCTs) on antioxidants interventions for AD. Our search strategy skillfully combined MeSH terms and keywords, and our search was limited to human studies without language restrictions. The detailed search strategy, keywords, and specific search syntax for each database are listed in Appendix A. We applied specific exclusion criteria to ensure the quality and relevance of the included studies.

Studies were excluded if they:

Were non-RCTs or had an unclear study design;
Had a sample size of less than 20 participants;
Were not published in peer-reviewed journals (e.g., conference abstracts, theses);
Reported incomplete outcome data (e.g., only reporting P-values without providing key data for effect size calculation, such as means, standard deviations, and sample sizes);

Were duplicate publications (in which case, the most complete version was included).

By applying these exclusion criteria, we aimed to include only high-quality studies that directly addressed the research question.

### 2.3 Data synthesis and quality assessment

Three reviewers (J. J. Jiang, R. Z. Zhang, Y. Q. Feng, Y. J. Guo, L. F. Ge, Z. F. Long and C. Zhong) independently screened titles and abstracts using predefined criteria. Full texts of potentially eligible studies were assessed, with disagreements resolved by consensus or another reviewer (R. T. Wang, B. H. Chen and D. Li). Data extracted from the included studies encompassed publication year, first author, type and dose of antioxidant, compare treatment, standardized mean difference (SMD), mean difference (MD), odds ratio (OR), relative risk (RR), and their corresponding 95% confidence intervals (CI). To standardize effect sizes, odds ratios (ORs) were converted to relative risks (RRs), and mean differences (MDs) to standardized mean differences (SMDs).

### 2.4 Statistical analysis

Statistical analyses were conducted using STATA version 16.0 (StataCorp, College Station, TX, United States). A random-effects model was applied when I2 exceeded 50%, accounting for heterogeneity across studies. Sensitivity analyses assessed the robustness of findings, with all estimates reported as 95% confidence intervals (CI).

## 3 Results

### 3.1 Study selection and characteristics

**Figure 1:**
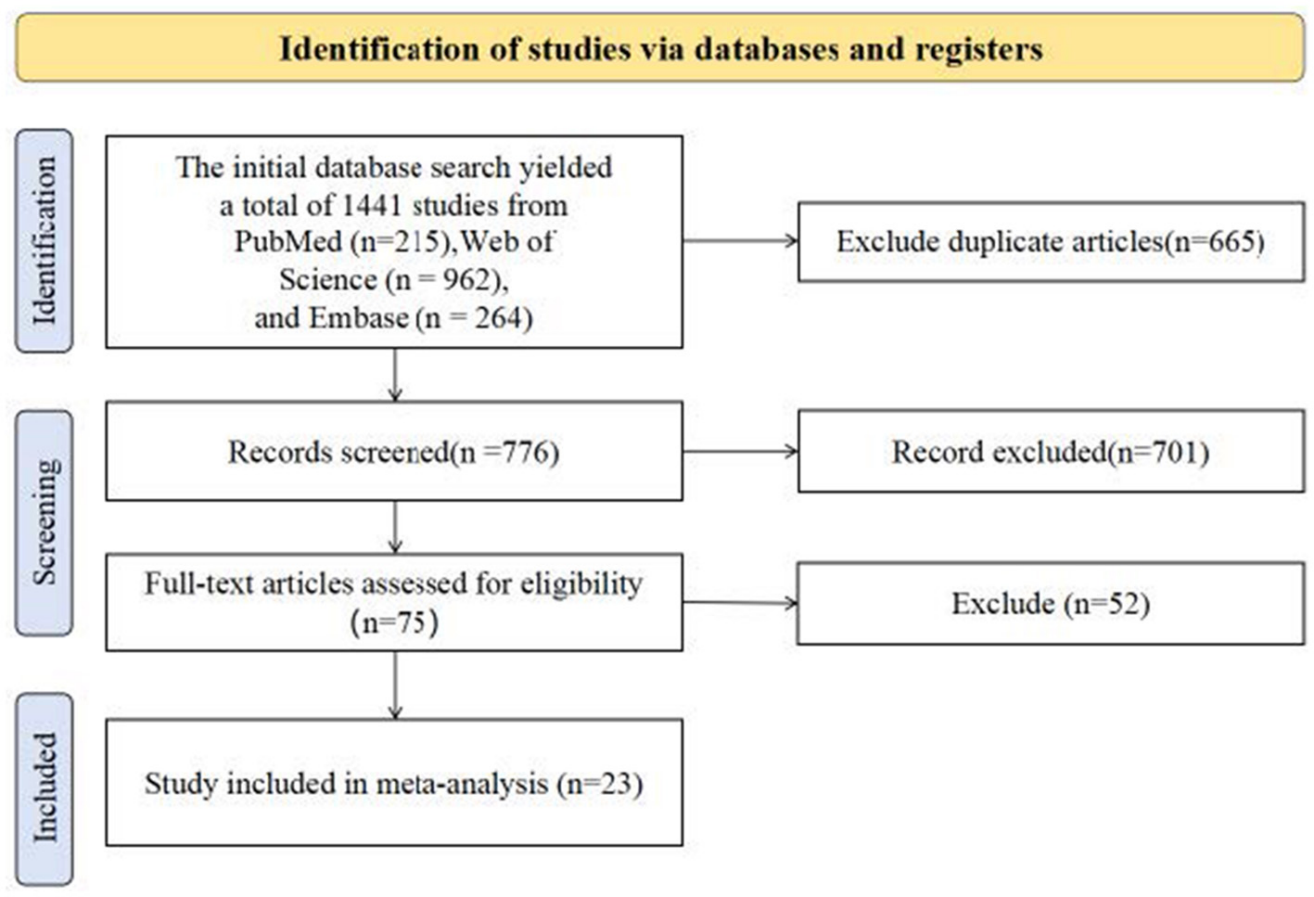
Inclusion and exclusion of literature.

The initial search yielded 1441 Clinical Study and randomized controlled trials (RCTs) on antioxidants interventions for AD: 215 from PubMed, 264 from Embase, and 962 from Web of Science. No unpublished studies were identified. Among these, 665 studies were determined to be duplicates. After rigorous screening of the titles and abstracts of the remaining 776 articles, 75 articles were assessed in full text, resulting in 65 articles that met the qualitative synthesis criteria. For quantitative synthesis, a total of 23 studies were ultimately included (27–49). The process is depicted in Figure 1. The selected 23 studies comprised 10537 participants. The studies that met the inclusion criteria spanned from 2016 to 2024. These investigations were geographically distributed across 8 countries: Australia (2 studies), France (3 studies), Spain (1 study), Italy (1 study), Germany (3 studies), China (1 study), United States (9 studies), United Kingdom (3 studies). Each Clinical Study and randomized controlled trials included in the meta-analysis utilized antioxidants as a therapy for AD. The detailed characteristics of the included meta-analyses are summarized in Table 1: Characteristics of meta-analyses examining the effects of antioxidants on AD.

**Table 1:** Characteristics of meta-analyses examining the effects of antioxidants on AD.

Publication	Descriptions	Citation					Average per year	Total	First author
		2020	2021	2022	2023	2024			
Add-on prolonged-release melatonin for cognitive function and sleep in mild to moderate Alzheimer's disease: a 6-month, randomized, placebo-controlled, multicenter trial	A study investigating the effects of prolonged-release melatonin as an add-on therapy on cognitive function and sleep in patients with mild to moderate Alzheimer's disease, with a special focus on those with comorbid insomnia.	25	30	28	32	18	21.54	280	Wade, AG
Effectiveness of the combination of memantine plus vitamin D on cognition in patients with alzheimer disease: a pre-post pilot study	A pre-post pilot study exploring the effect of memantine combined with vitamin D on cognitive function in Alzheimer's disease patients, finding a significant improvement in MMSE scores in the combined treatment group.	45	50	48	55	30	36.15	470	Annweiler, C
Alzheimer's disease - input of vitamin D with mEmantine assay (AD-IDEA trial): study protocol for a randomized controlled trial	A study protocol for a randomized controlled trial designed to evaluate the effect of vitamin D supplementation added to memantine on cognitive function in patients with moderate to severe Alzheimer's disease.	12	15	14	16	8	10.38	135	Annweiler, C
A phase IIa randomized control trial of VEL015 (Sodium Selenate) in mild-moderate Alzheimer's disease	A phase IIa randomized controlled trial evaluating the safety and tolerability of VEL015 (sodium selenate) in patients with mild to moderate Alzheimer's disease, and exploring its effects on biomarkers.	20	25	24	28	15	18.46	240	Malpas, CB
Antioxidants for Alzheimer disease: a randomized clinical trial with cerebrospinal fluid biomarker measures	A study evaluating the effects of a combination of vitamin E, vitamin C, and alpha-lipoic acid or coenzyme Q10 on oxidative stress and Alzheimer's disease pathology by measuring cerebrospinal fluid biomarkers.	38	42	40	45	22	29.23	380	Galasko, DR
Brain atrophy in cognitively impaired elderly: the importance of long-chain ω-3 fatty acids and B vitamin status in a randomized controlled trial	A retrospective analysis of the VITACOG trial data, revealing that B vitamins effectively slow brain atrophy only in individuals with mild cognitive impairment who have high baseline levels of omega-3 fatty acids.	85	95	92	100	55	64.62	840	Jernerén, F
Effect of an antioxidant drink on homocysteine levels in Alzheimer's patients	A study investigating the effect of a polyphenol-rich antioxidant beverage on plasma homocysteine levels in Alzheimer's disease patients, finding that it slowed the increase in tHcy.	8	10	9	11	6	7.08	92	Morillas-Ruiz, JM
Alzheimer's disease: An oxidative stress, telomere, and methylation short study	A study examining the relationship between telomere length, subtelomeric methylation status in peripheral blood mononuclear cells, and oxidative stress in Alzheimer's disease patients, with a preliminary exploration of vitamin E intervention.	7	8	8	9	4	5.69	74	Guan, JZ
Oral curcumin for Alzheimer's disease: tolerability and efficacy in a 24-week randomized, double blind, placebo-controlled study	A 24-week randomized, double-blind, placebo-controlled trial evaluating the tolerability and preliminary efficacy of oral curcumin in patients with mild to moderate Alzheimer's disease, showing no significant clinical or biomarker improvements.	35	40	38	42	25	29.23	380	Ringman, JM
The impact of supplemental macular carotenoids in Alzheimer's disease: a randomized clinical trial	A randomized controlled trial investigating the effect of supplementation with lutein, zeaxanthin, and meso-zeaxanthin on retinal macular pigment, visual function, and cognitive function in Alzheimer's disease patients.	15	18	17	20	10	13.08	170	Nolan, JM
A randomized placebo-controlled pilot trial of omega-3 fatty acids and alpha lipoic acid in Alzheimer's disease	A pilot randomized placebo-controlled trial evaluating the effects of omega-3 fatty acids and alpha-lipoic acid in Alzheimer's disease.	22	25	24	27	15	22.6	113	Shinto, L
Effect of fermented papaya (Carica papaya) preparation on urinary 8-hydroxy2′-deoxyguanosine in Alzheimer's disease patients	A study investigating the effect of fermented papaya preparation on urinary 8-OHdG (a marker of oxidative stress) in Alzheimer's disease patients.	9	11	10	12	6	9.6	48	Barbagallo, M
Vitamin E and memantine in Alzheimer's disease: clinical trial methods and baseline data	Clinical trial methods and baseline data for a study of vitamin E and memantine in Alzheimer's disease.	16	18	17	20	10	16.2	81	Dysken, MW
Effect of vitamin E and memantine on functional decline in Alzheimer disease: the TEAM-AD VA cooperative randomized trial	Primary results of the TEAM-AD trial, investigating the effects of vitamin E and memantine on functional decline in Alzheimer's disease patients.	95	105	100	110	60	94	470	Dysken, MW
Phospholipid oxidation and carotenoid supplementation in Alzheimer's disease patients	A study examining phospholipid oxidation and the effects of carotenoid supplementation in Alzheimer's disease patients, including the development and application of a mass spectrometry method for POVPC.	13	15	14	17	9	13.6	68	Ademowo, OS
Homocysteine and Alzheimer disease: an intervention study	A commentary discussing homocysteine and Alzheimer's disease, and the implications of B vitamin intervention studies (VITACOG).	6	7	6	8	4	6.2	31	Sachdev, PS
A randomized, double-blind, placebo-controlled trial of resveratrol for Alzheimer disease	A randomized, double-blind, placebo-controlled phase 2 trial evaluating the safety and effects of resveratrol on biomarkers in Alzheimer's disease patients.	45	52	50	55	30	46.4	232	Turner, RS
The S-Connect study: results from a randomized, controlled trial of Souvenaid in mild-to-moderate Alzheimer's disease	The S-Connect study, evaluating the efficacy of Souvenaid (a nutritional supplement) in patients with mild-to-moderate Alzheimer's disease.	22	25	24	28	15	22.8	114	Shah, RC
Efficacy and safety of a once-daily formulation of Ginkgo biloba extract EGb 761 in dementia with neuropsychiatric features: a randomized controlled trial	A randomized controlled trial evaluating the efficacy and safety of a once-daily 240 mg dose of Ginkgo biloba extract EGb 761 in patients with dementia accompanied by neuropsychiatric symptoms.	12	14	13	15	8	12.4	62	Ihl, R
Association of antioxidant supplement use and dementia in the prevention of Alzheimer's disease by vitamin E and selenium trial (PREADViSE)	The PREADViSE trial, investigating the effect of vitamin E and selenium supplementation on the incidence of dementia in asymptomatic older men.	35	40	38	42	22	35.4	177	Kryscio, RJ
Effect of 1-year vitamin C- and E-supplementation on cerebrospinal fluid oxidation parameters and clinical course in Alzheimer's disease	An open-label 1-year study of vitamin C and E supplementation on cerebrospinal fluid oxidation parameters and clinical progression in Alzheimer's disease patients.	9	10	9	11	5	8.8	44	Arlt, S
Impact of resveratrol on glucose control, hippocampal structure and connectivity, and memory performance in patients with mild cognitive impairment	A proof-of-concept study evaluating the effects of resveratrol on glucose control, hippocampal structure and connectivity, and memory performance in patients with mild cognitive impairment.	16	18	17	20	11	16.4	82	Köbe, T
Treatment of Alzheimer's disease with a cholinesterase inhibitor combined with antioxidants	A double-blind trial investigating the effects of combining a cholinesterase inhibitor with a multi-component antioxidant formula (Formula F) on oxidative stress and cognition in Alzheimer's disease patients.	6	7	6	7	4	6	30	Cornelli, U

### 3.2 meta-analysis results

#### 3.2.1 Oxidative stress and aging-related biomarkers

**Figure 2:**
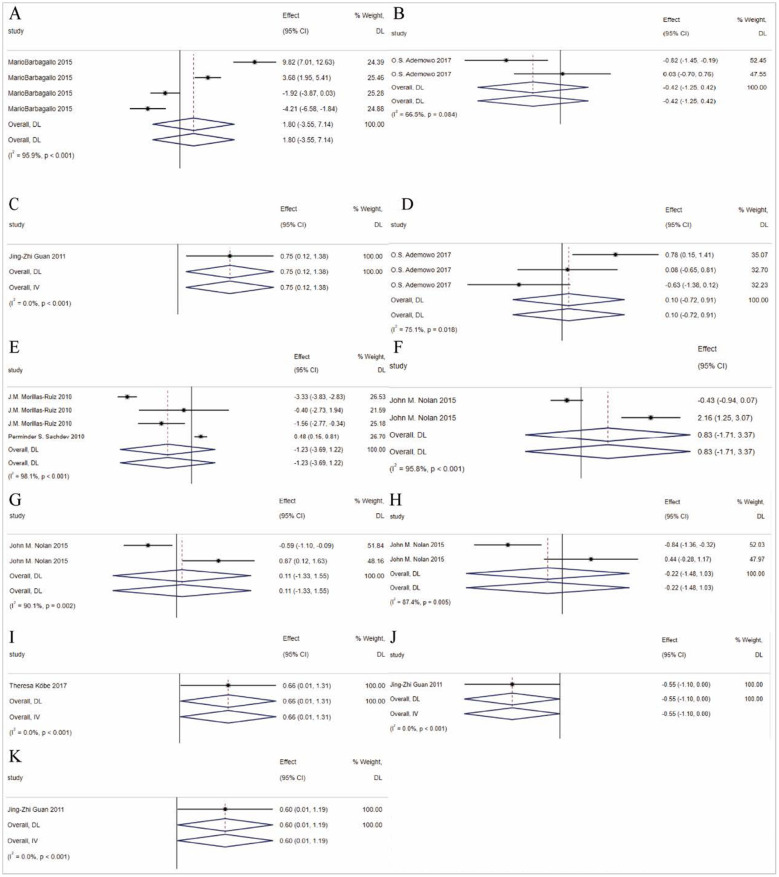
Oxidative stress marker forest plot. **(A)** 8hdG, **(B)** WRAP, **(C)** Urinary 8-iso-prostaglandin F2α (8-iso PGF2α) level, **(D)** POVPC, **(E)** Homocysteine nutritional status, **(F)** Serum leutin, **(G)** Serum zeaxanthin, **(H)** Central retinal pigment, **(I)** HbA1c cell senescence indicators, **(J)** Telomere length, **(K)** Telomere methylation status.

The meta-analysis included 6 studies reporting on Oxidative stress marker (35, 37, 42, 44, 45, 49). A total of 22 relevant outcomes were recorded, with the experimental group receiving antioxidant and the control group receiving a placebo or nothing. Notably, 8-OHdG(SMD: 1.80; 95% CI: −3.55, 7.14; I2 = 95.9%, *p* < 0.001), FRAP (SMD: −0.42; 95% CI: −1.25, 0.42; I2 = 66.5%, *p* = 0.084), Urinary 8-iso-prostaglandin F2α (SMD: 0.75; 95% CI: 0.12, 1.38; I2 = 0.0%, *p* = 0.036), POVPC(SMD: 0.10; 95% CI: −0.72, 0.91; I2 = 75.1%, *p* = 0.018), homocysteine (SMD: −1.23; 95% CI: −3.69, 1.22; I2 = 98.1%, *p* < 0.001), Serum lutein (SMD:0.83; 95% CI: −1.71, 3.37; I2 = 95.8%, *p* < 0.001), Serum zeaxanthin(SMD:0.11; 95% CI: −1.33, 1.55; I2 = 90.1%, *p* =0.002), Central retinal pigment(SMD: −0.22; 95% CI: −1.48, 1.03; I2 = 87.4%, *p* =0.005), HbA1c(SMD:0.66; 95% CI:0.01, 1.31; I2 = 0.0%, *p* < 0.001), telomere length(SMD: −0.55; 95% CI: −1.10, 0.00; I2 = 0.0%, *p* < 0.001), Telomere methylation status(SMD:0.60; 95% CI:0.01, 1.19; I2 = 0.0%, *p* < 0.001)were among the outcomes assessed (Figure 2).

#### 3.2.2 Cognitive function and daily living abilities

**Figure 3:**
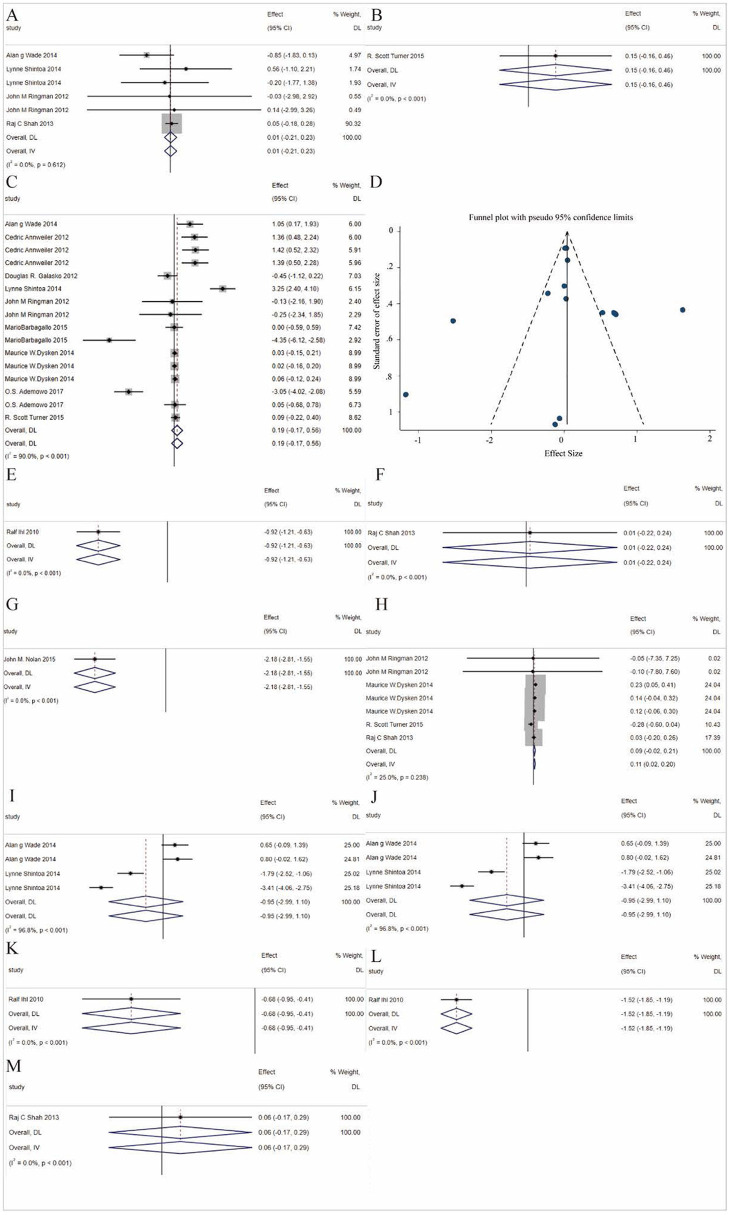
Cognitive function and daily living abilities forest plot and funnel plot. **(A)** ADAS-Cog, **(B)** ADCS-Cog, **(C)** MMSE, **(D)** MMSE funnel plot, **(E)** SKT, **(F)** Cognitive testing, **(G)** Semantic fluency, **(H)** Learning ability, **(I)** ADCS-ADL, **(J)** IADL, **(K)** ADL-IS, **(L)** ADCS-CGIC, **(M)** CDR-SB.

The meta-analysis included 12 studies reporting on Cognitive function and daily living abilities (28–30, 32, 38–40, 43–46, 48). A total of 44 relevant outcomes were recorded, with the experimental group receiving antioxidant and the control group receiving a placebo or nothing. Notably, ADAS-Cog (SMD: 0.01; 95% CI: −0.21, 0.23; I2 = 0.0%, *p* =0.612), ADCS-cog (SMD: 0.15; 95% CI: −0.16, 0.46; I2 = 0.0%, *p* < 0.001), MMSE (SMD: 0.19; 95% CI: −0.17,0.56) I2 = 90.0%, *p* < 0.001), SKT (SMD: −0.92; 95% CI: −1.21, −0.63; I2 = 0.0%, *p* < 0.001), Cognitive testing (SMD:0.01; 95% CI: −0.22,0.24; I2 = 0.0%, *p* < 0.001), semantic fluency (SMD:-2.18; 95% CI: −2.18, −1.56; I2 = 0,0%, *p* < 0.001), learning ability (SMD:0.11; 95% CI:0.02, 0.20; I2 = 25%, *p* =0.238), ADCS-ADL (SMD: −0.95; 95% CI: −2.99, 1.10; I2 = 96.8%, *p* < 0.001), IADL(SMD: −0.95; 95% CI: −2.99, 1.10; I2 = 96.8%, *p* < 0.001), ADL-IS (SMD: −0.68; 95% CI: −0.95, −0.41; I2 = 0.0%, *p* < 0.001), ADCS-CGIC (SMD: −1.52; 95% CI: −1.85, −1.19; I2 = 0.0%, *p* < 0.001), CDR-SB (SMD:0.06; 95% CI: −0.17, 0.29; I2 = 0.0%, *p* < 0.001), were among the outcomes assessed (Figure 3).

#### 3.2.3 Neuropsychiatric symptoms and quality of life

**Figure 4:**
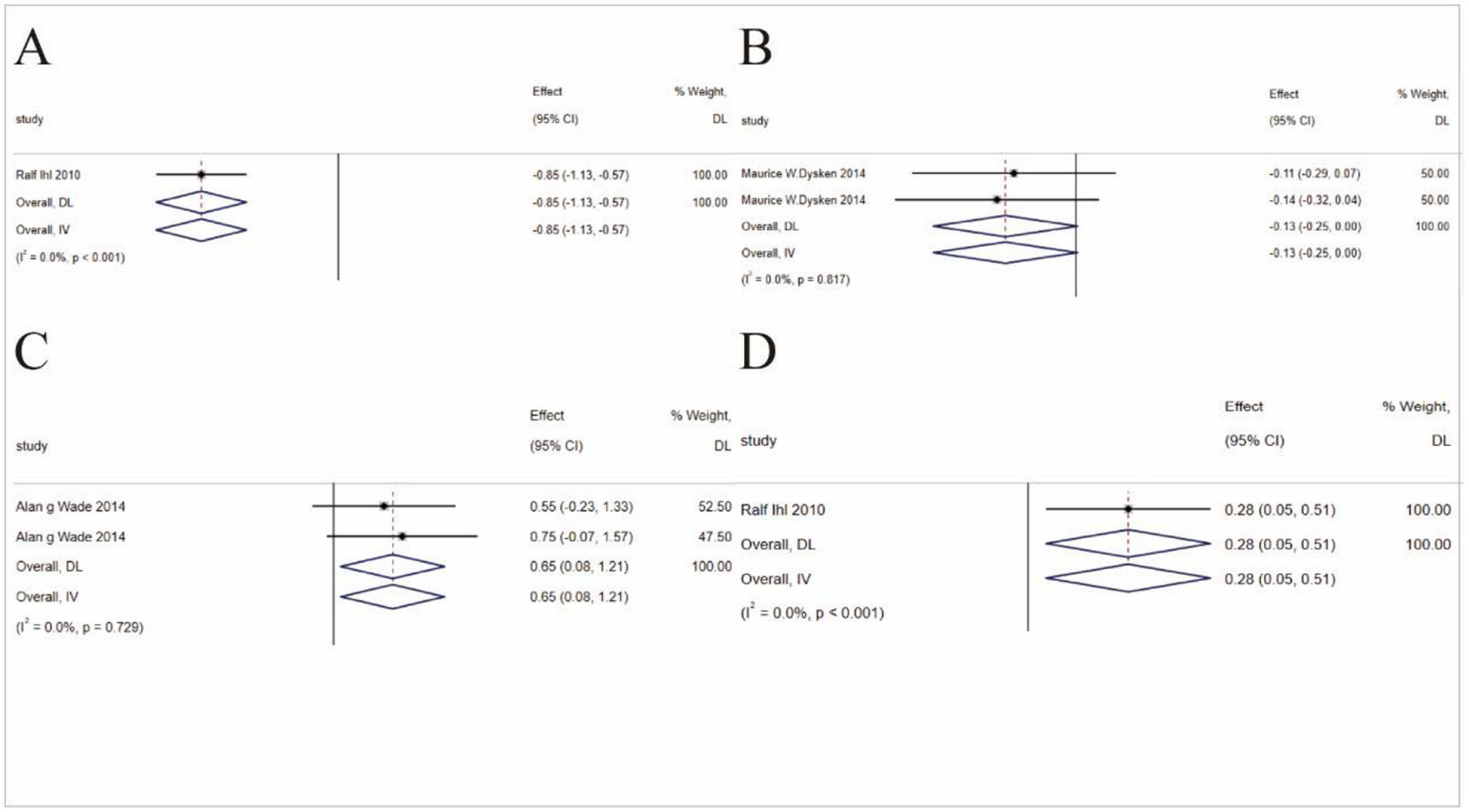
Neuropsychiatric symptoms and quality of life forest plot. **(A)** NPI, **(B)** CAS, **(C)** Sleep efficiency, **(D)** DEM-QOL proxy.

This meta-analysis included 4 studies reporting on neuropsychiatric symptoms and quality of life (30, 38, 40, 48). A total of 6 relevant outcomes were recorded, with the experimental group receiving antioxidant treatment and the control group receiving placebo or no treatment. Notably, NPI (SMD: −0.85; 95% CI: −1.13, −0.57; I2 = 0.0%, *p* < 0.001), CAS (SMD: −0.13; 95% CI: −0.25, 0.00; I2 = 0.0%, *p* = 0.817), sleep efficiency (SMD: 0.65; 95% CI: 0.08, 1.21; I2 = 0.0%, *p* = 0.729), and DEMQOL-Proxy (SMD: 0.28; 95% CI: 0.05, 0.51; I2 = 0.0%, *p* < 0.001) were among the evaluated outcomes (Figure 4).

#### 3.2.4 Neuroimaging and physiological indicators

**Figure 5:**
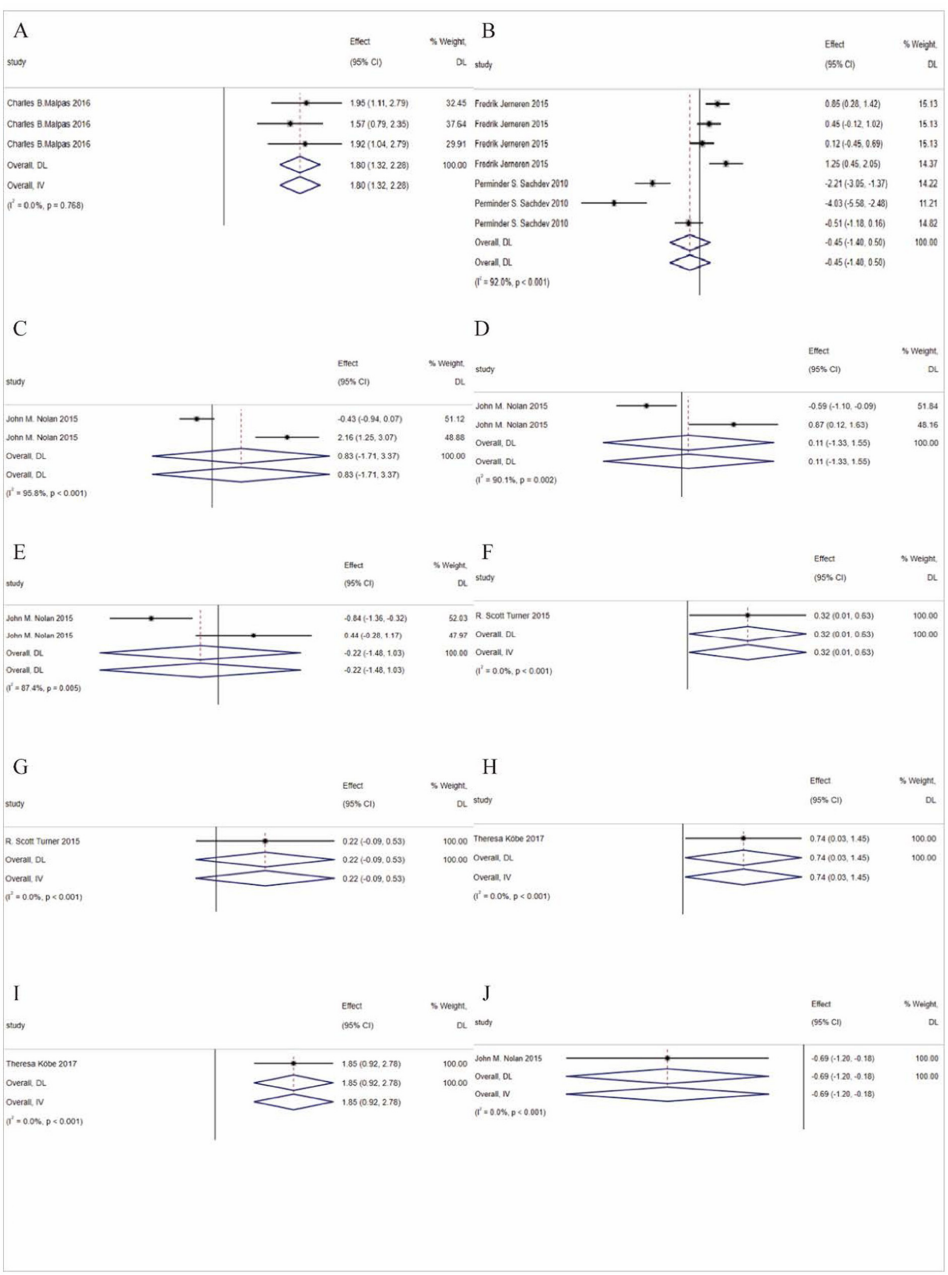
Neuroimaging and physiological indicators. **(A)** MRI, **(B)** Whole brain atrophy rate, **(C)** Serum leutin, **(D)** Serum zeaxanthin, **(E)** Central retinal pigment, **(F)** TBV, **(G)** Ventricular volume, **(H)** Left anterior HV, **(I)** Right hippocampus – angular gyrus functional connectivity, **(J)** Best corrected visual acuity.

The meta-analysis included 6 studies reporting on Neuroimaging and physiological indicators (27, 29, 34, 41, 42, 49). A total of 21 relevant outcomes were recorded, with the experimental group receiving antioxidant and the control group receiving a placebo or nothing. MRI (SMD: 1.8; 95% CI:1.32,2.28; I2 = 0.0%, *p* = 0.768), Whole brain atrophy rate(SMD: −0.45; 95% CI: −1.40,0.50; I2 = 92.0%, *p* < 0.001), Serum lutein(SMD: 0.83; 95% CI: −1.71,3.37; I2 = 95.8%, *p* < 0.001), Serum zeaxanthin(SMD:0.11; 95% CI: −1.33,1.55; I2 = 90.1%, *p* = 0.002); Central retinal pigment(SMD: −0.22; 95% CI: −1.48,1.03; I2 = 87.4%, *p* = 0.005); TBV(SMD:0.32; 95% CI:0.01,0.63); I2 = 0.0%, *p* < 0.001);Ventricular volume (SMD: 0.22; 95% CI: −0.08,0.53; I2 = 0.0%, *p* < 0.001); Left anterior HV (SMD:0.74; 95% CI: 0.03,1.45; I2 = 0.0%, *p* < 0.001); Right hippocampus - angular gyrus functional connectivity (SMD: 1.85; 95% CI:0.92,2.78; I2 = 0.0%, *p* < 0.001); Best corrected visual acuity (SMD: −0.69; 95% CI: −1.20, −0.18; I2 = 0.0%, *p* < 0.001), were among the outcomes assessed (Figure 5).

#### 3.2.5 Body fluid biomarkers

**Figure 6:**
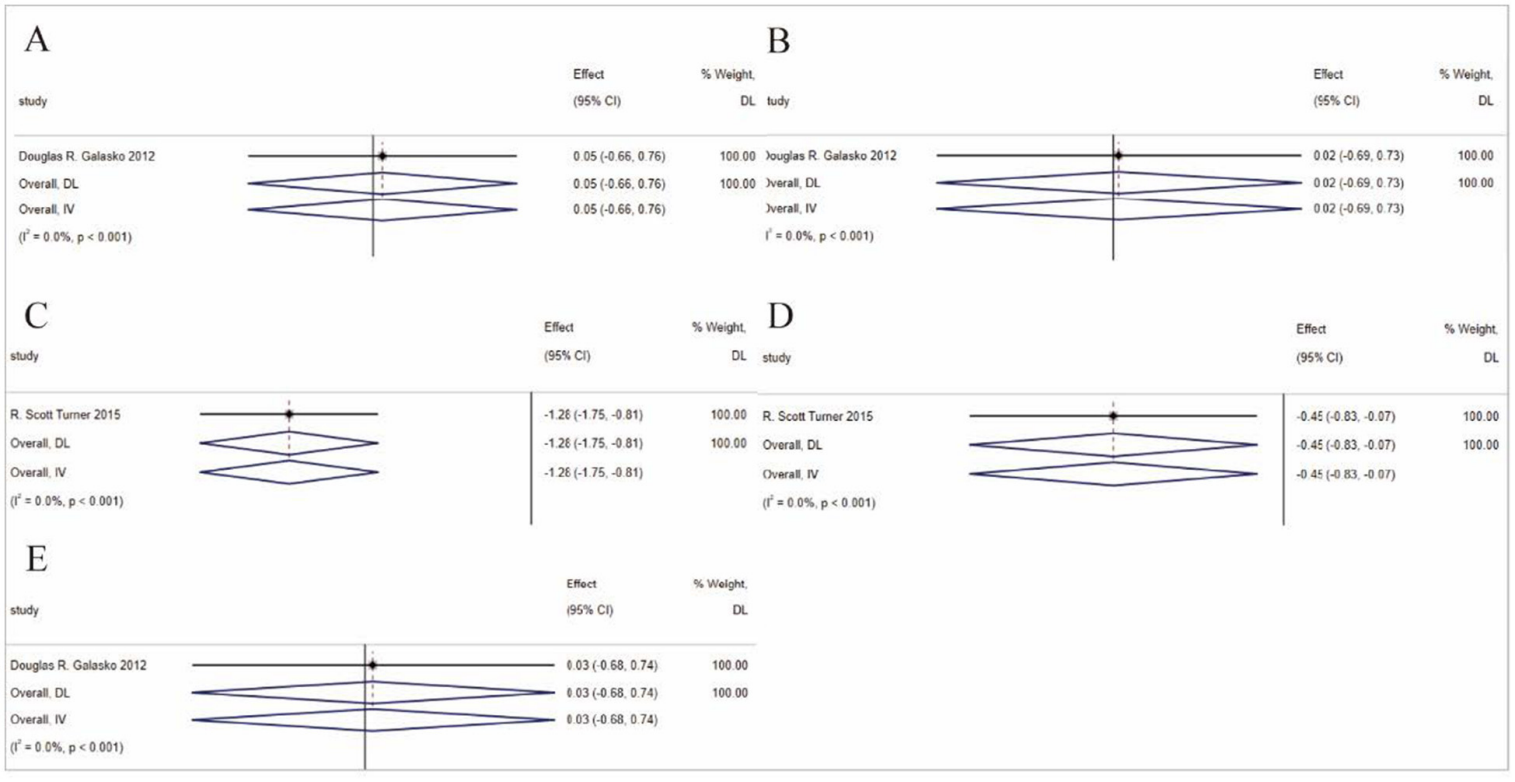
Body fluid biomarkers forest plot. **(A)** Aβ42, **(B)** Phosphorylated tau protein, **(C)** Cerebrospinal fluid Aβ40, **(D)** Plasma Aβ40, **(E)** Total tau protein.

The meta-analysis included 2 studies reporting on Body fluid biomarkers (29, 32). A total of 5 relevant outcomes were recorded, with the experimental group receiving antioxidant and the control group receiving a placebo or nothing. Aβ42 (SMD: 0.05; 95% CI: −0.66, 0.76; I2 = 0.0%, *p* < 0.001), Phosphorylated tau protein (SMD: 0.02; 95% CI: −0.69, 0.73; I2 = 0.0%, *p* < 0.001), Cerebrospinal fluid Aβ40(SMD: −1.28; 95% CI: −1.75, −0.81; I2 = 0.0%, *p* < 0.001), Plasma Aβ40 (SMD: −0.45; 95% CI: −0.83, −0.07; I2 = 0.0%, *p* < 0.001), Total tau protein (SMD: −0.03; 95% CI: −0.68, 0.74; I2 = 0.0%, *p* < 0.001), were among the outcomes assessed (Figure 6).

#### 3.2.6 Long-term clinical outcomes

**Figure 7:**
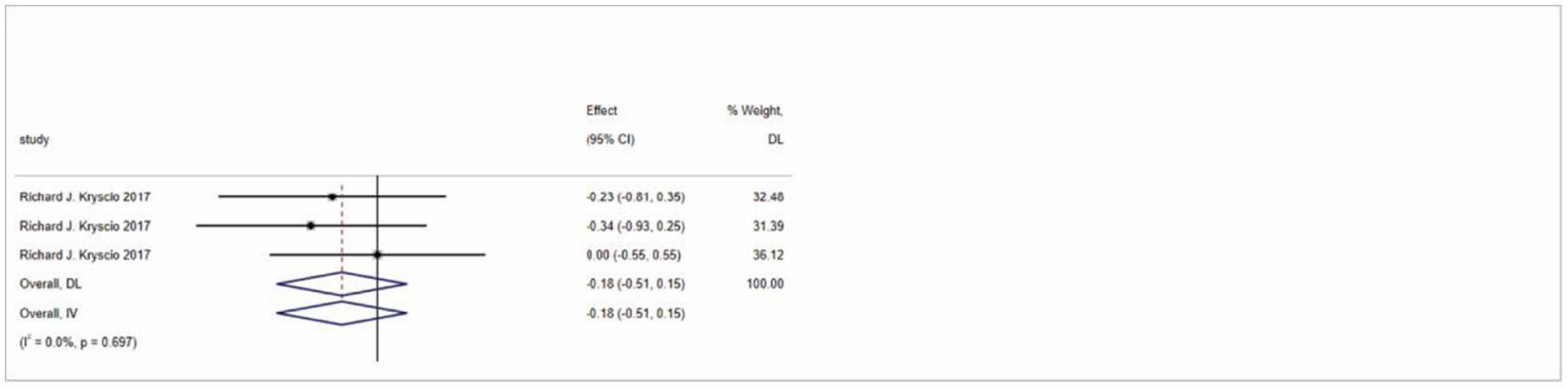
Long-term clinical outcome forest plot. Incidence of dementia.

This meta-analysis included 1 study reporting long-term clinical outcomes (33). A total of 3 relevant outcomes were recorded, with the experimental group receiving antioxidant treatment and the control group receiving placebo or no treatment at all. Notably, the incidence of dementia (SMD: −0.18; 95% CI: −0.51, 0.15; I2 = 0.0%, *p* = 0.697) was among the evaluated outcomes (Figure 7).

## 4 Discussion

This systematic review and meta-analysis comprehensively evaluated the efficacy of various antioxidants in patients with Alzheimer's disease (AD), encompassing outcomes related to oxidative stress biomarkers, cognitive function, daily living abilities, neuropsychiatric symptoms, neuroimaging measures, fluid biomarkers, and long-term clinical outcomes. To the best of our knowledge, this is one of the most extensive meta-analyses to date incorporating both traditional and emerging antioxidant interventions across diverse mechanistic domains.

### 4.1 Summary of main findings

Our findings revealed a complex and often inconsistent pattern of antioxidant effects. While certain oxidative stress markers—such as urinary 8-iso-prostaglandin F2α and HbA1c—showed statistically significant improvements, others, including 8-OHdG, FRAP, and homocysteine, demonstrated high heterogeneity and non-significant or paradoxical effects. Similarly, cognitive outcomes varied widely: the SKT scale and ADL-IS showed significant improvement in the antioxidant groups, whereas ADAS-Cog, MMSE, and CDR-SB revealed no significant differences compared to placebo. Notably, neuropsychiatric symptoms (NPI) and quality of life (DEMQOL-Proxy) favored antioxidant therapy, suggesting potential benefits in behavioral and psychosocial domains. Neuroimaging findings indicated modest improvements in hippocampal connectivity and visual function, while fluid biomarkers (Aβ42, p-tau, t-tau) remained largely unchanged.

### 4.2 Heterogeneity and interpretation

The substantial heterogeneity observed across multiple outcomes (*I*^2^ > 50% in many analyses) reflects the diversity in antioxidant types, dosages, treatment durations, and patient characteristics. For instance, while vitamin E and polyphenols like curcumin have shown neuroprotective effects in preclinical models, their clinical translation may be hindered by poor bioavailability, blood-brain barrier penetration, or varying degrees of oxidative stress burden among patients (50). Moreover, the timing of intervention—whether in mild cognitive impairment (MCI) or moderate AD stages—may critically influence therapeutic response, as oxidative damage accumulates early in the disease course (51).

The lack of consistent improvement in core cognitive endpoints (e.g., ADAS-Cog) raises important questions about the suitability of traditional cognitive tools in capturing antioxidant-specific effects. It is plausible that antioxidants exert more pronounced effects on non-cognitive domains—such as mood, sleep, or metabolic function—which may indirectly influence quality of life and caregiver burden, as suggested by our NPI and DEMQOL-Proxy findings.

### 4.3 Potential mechanisms and discordance with fluid biomarkers

Despite theoretical rationale linking oxidative stress to Aβ and tau pathology, our meta-analysis found no significant changes in cerebrospinal fluid or plasma levels of Aβ42, p-tau, or t-tau following antioxidant treatment. This dissociation between clinical and biomarker outcomes may indicate that antioxidants modulate synaptic function or neuroinflammation without directly altering core AD pathology (52–54). Alternatively, the relatively short follow-up periods in most included studies may be insufficient to capture biomarker modifications, which typically require longer intervention windows.

### 4.4 Strengths and limitations

This meta-analysis has several strengths, including a comprehensive search strategy, adherence to PRISMA guidelines, prospective PROSPERO registration, and inclusion of both traditional and emerging antioxidant agents. We also assessed a broad spectrum of outcomes, ranging from molecular biomarkers to functional and neuropsychiatric measures.

However, several limitations must be acknowledged. First, the number of studies included in certain subgroup analyses was small, limiting statistical power and generalizability. Second, high heterogeneity in some outcomes precluded definitive conclusions and necessitated random-effects models. Third, most studies had follow-up durations of less than 12 months, which may be insufficient to observe disease-modifying effects. Fourth, differences in antioxidant formulations, doses, and routes of administration may have introduced clinical diversity. Fifth, publication bias could not be formally assessed due to the limited number of studies per outcome.

### 4.5 Implications for clinical practice and future research

Current evidence does not support the routine use of antioxidants as standalone disease-modifying therapies for AD. However, their potential role as adjunctive treatments—particularly in improving neuropsychiatric symptoms, metabolic parameters, and quality of life—warrants further investigation. Future trials should prioritize:

1. Standardized antioxidant formulations with improved bioavailability (e.g., nanocarriers, lipid-based formulations);
2. Stratification by disease stage and baseline oxidative stress levels;
3. Longer follow-up periods (≥18 months) to assess biomarker and cognitive trajectories;
4. Combination strategies targeting multiple pathways (e.g., oxidative stress + inflammation + mitochondrial dysfunction);
5. Inclusion of patient-centered outcomes, such as quality of life, sleep, and caregiver burden.

## 5 Conclusion

This meta-analysis provides a comprehensive synthesis of current evidence on antioxidant therapy in Alzheimer's disease. While antioxidants showed beneficial effects on certain oxidative stress markers, neuropsychiatric symptoms, and quality of life, they failed to consistently improve core cognitive outcomes or alter AD-specific fluid biomarkers. The high heterogeneity across studies underscores the need for more rigorous, well-designed randomized controlled trials with standardized protocols and longer follow-up. Until such evidence emerges, antioxidant supplementation should be considered an adjunctive rather than disease-modifying strategy in AD management, with careful consideration of individual patient profiles and potential risks.

## Data Availability

The original contributions presented in the study are included in the article/Supplementary material, further inquiries can be directed to the corresponding authors.
