# Supplementary material for: Efficacy of antioxidants as a therapy for Alzheimer's disease: a meta-analysis
Source: Front Nutr. 2026 Jun 23;13:1878597. doi: 10.3389/fnut.2026.1878597 (PMC13337380; doi:10.3389/fnut.2026.1878597)
Supplement: Supplementary file 1 [file Table_1.docx]

**Search term**

**Appendix 1. Pubmed search strategy**

**Searched 2025/8/21**

#1 Alzheimer Disease[MeSH Terms] (135,163)

#2 (((((((((((((((((((((((((((((((((((Alzheimer Disease[Title/Abstract]) OR (Alzheimer Syndrome[Title/Abstract])) OR (Alzheimer-Type Dementia (ATD[Title/Abstract]))) OR (Alzheimer Type Dementia (ATD[Title/Abstract]))) OR (Dementia, Alzheimer-Type (ATD[Title/Abstract]))) OR (Alzheimer's Diseases[Title/Abstract])) OR (Alzheimer Diseases[Title/Abstract])) OR (Alzheimers Diseases[Title/Abstract])) OR (Alzheimer Dementia[Title/Abstract])) OR (Alzheimer Dementias[Title/Abstract])) OR (Dementia, Alzheimer[Title/Abstract])) OR (Alzheimer's Disease[Title/Abstract])) OR (Dementia, Senile[Title/Abstract])) OR (Senile Dementia[Title/Abstract])) OR (Dementia, Alzheimer Type[Title/Abstract])) OR (Alzheimer Type Dementia[Title/Abstract])) OR (Senile Dementia, Alzheimer Type[Title/Abstract])) OR (Alzheimer Type Senile Dementia[Title/Abstract])) OR (Primary Senile Degenerative Dementia[Title/Abstract])) OR (Alzheimer Sclerosis[Title/Abstract])) OR (Sclerosis, Alzheimer[Title/Abstract])) OR (Dementia, Primary Senile Degenerative[Title/Abstract])) OR (Dementia, Presenile[Title/Abstract])) OR (Presenile Dementia[Title/Abstract])) OR (Acute Confusional Senile Dementia[Title/Abstract])) OR (Senile Dementia, Acute Confusional[Title/Abstract])) OR (Alzheimer Disease, Early Onset[Title/Abstract])) OR (Early Onset Alzheimer Disease[Title/Abstract])) OR (Presenile Alzheimer Dementia[Title/Abstract])) OR (Alzheimer Disease, Late Onset[Title/Abstract])) OR (Late Onset Alzheimer Disease[Title/Abstract])) OR (Alzheimer's Disease, Focal Onset[Title/Abstract])) OR (Focal Onset Alzheimer's Disease[Title/Abstract])) OR (Familial Alzheimer Disease (FAD[Title/Abstract]))) OR (Alzheimer Disease, Familial (FAD[Title/Abstract]))) OR (Familial Alzheimer Diseases (FAD[Title/Abstract])) (207,366)

#3 #1 OR #2 (225,646)

#4 "antioxidants"[MeSH Terms] OR "vitamins"[MeSH Terms] OR "vitamin e"[MeSH Terms] OR "ascorbic acid"[MeSH Terms] OR "tocopherols"[MeSH Terms] OR "selenium"[MeSH Terms] OR "zinc"[MeSH Terms] OR "ubiquinone"[MeSH Terms] OR "acetylcysteine"[MeSH Terms] OR "carnitine"[MeSH Terms] OR "melatonin"[MeSH Terms] OR "glutathione"[MeSH Terms] OR "carotenoids"[MeSH Terms] OR "arginine"[MeSH Terms] OR "resveratrol"[MeSH Terms] OR "vitamin d"[MeSH Terms] OR "ergocalciferols"[MeSH Terms] OR "curcumin"[MeSH Terms] (695,904)

#5 Antioxidants"[Title/Abstract] OR "Vitamin"[Title/Abstract] OR "vitamin e"[Title/Abstract] OR "tocopherol*"[Title/Abstract] OR "alpha tocopherol*"[Title/Abstract] OR "tocotrienol"[Title/Abstract] OR "vitamin c"[Title/Abstract] OR "ascorbic acid"[Title/Abstract] OR "ascorb*"[Title/Abstract] OR "selenium"[Title/Abstract] OR "selen*"[Title/Abstract] OR "zinc"[Title/Abstract] OR "zinc*"[Title/Abstract] OR "ubiquinone"[Title/Abstract] OR "ubiquinol"[Title/Abstract] OR "coenzyme q10"[Title/Abstract] OR "CoQ10"[Title/Abstract] OR "Acetylcysteine"[Title/Abstract] OR "Carnitine"[Title/Abstract] OR "carnitene"[Title/Abstract] OR "melatonin"[Title/Abstract] OR "Glutathione"[Title/Abstract] OR "GSH"[Title/Abstract] OR "carotene"[Title/Abstract] OR "betacarotene"[Title/Abstract] OR "Arginine"[Title/Abstract] OR "resveratrol*"[Title/Abstract] OR primrose"[Title/Abstract] "Curcumin"[Title/Abstract] OR "evening OR "evening primrose oil"[Title/Abstract] OR "vitamin d"[Title/Abstract] (87,363)

#6 #4 or #5 (730,981)

#7 #3 and #6 Filters: Clinical Study, Clinical Trial, Controlled Clinical Trial, Randomized Controlled Trial (215)

**Appendix 2. Embase search strategy**

**Searched 2025/8/21**

#1 'alzheimer disease':ab,ti OR 'alzheimer syndrome':ab,ti OR 'alzheimer-type dementia':ab,ti OR 'dementia, alzheimer-type':ab,ti OR 'alzheimer diseases':ab,ti OR 'alzheimers diseases':ab,ti OR 'alzheimer dementia':ab,ti OR 'alzheimer dementias':ab,ti OR 'dementia, alzheimer':ab,ti OR 'senile dementia':ab,ti OR 'dementia, alzheimer type':ab,ti OR 'alzheimer type dementia':ab,ti OR 'dementia, presenile':ab,ti OR 'senile dementia, acute confusional':ab,ti OR 'alzheimer disease, early onset':ab,ti OR 'familial alzheimer diseases':ab,ti (34,485)

#2 'antioxidants'/exp OR 'vitamin OR 'antioxidants' OR 'vitamins'/exp e' OR 'ascorbic acid'/exp OR 'vitamins' OR 'vitamin OR 'ascorbic e'/exp acid' OR 'tocopherols'/exp OR 'tocopherols' OR 'selenium'/exp OR 'selenium' OR 'zinc'/exp OR 'zinc' OR 'ubiquinone'/exp OR 'ubiquinone' OR 'acetylcysteine'/exp OR 'acetylcysteine' OR 'carnitine'/exp OR 'carnitine' OR 'melatonin'/exp OR 'glutathione' OR 'carotenoids'/exp OR 'melatonin' OR 'glutathione'/exp OR 'carotenoids' OR 'arginine'/exp OR 'arginine' OR 'resveratrol'/exp OR 'resveratrol' OR 'vitamin d'/exp OR 'vitamin d' OR 'evening primrose'/exp OR 'evening primrose' OR 'curcumin'/exp OR 'curcumin' 'antioxidants':ab,ti OR 'vitamin':ab,ti OR 'vitamin e':ab,ti OR 'tocopherol*':ab,ti OR 'alpha tocopherol*':ab,ti OR 'tocotrienol':ab,ti OR 'vitamin c':ab,ti OR 'ascorbic acid':ab,ti OR 'ascorb*':ab,ti OR 'selenium':ab,ti OR 'selen*':ab,ti OR 'zinc c':ab,ti OR 'zinc*':ab,ti OR 'ubiquinone':ab,ti OR 'ubiquinol':ab,ti OR 'acetylcysteine':ab,ti OR 'carnitine':ab,ti OR 'coenzyme q10':ab,ti OR 'carnitene':ab,ti OR 'coq10':ab,ti OR 'melatonin':ab,ti OR 'glutathione':ab,ti OR 'gsh':ab,ti OR 'carotene':ab,ti OR 'betacarotene':ab,ti OR 'arginine':ab,ti OR 'resveratrol*':ab,ti OR 'vitamin d':ab,ti OR 'curcumin':ab,ti OR 'evening primrose oil':ab,ti(1882,436)

#3 #1 and #2 (264)

**Appendix 3. Web of Science search strategy**

**Searched 2025/8/21**

#1 Alzheimer Disease (Topic) or Alzheimer Syndrome (Topic) or Alzheimer-Type Dementia (Topic) or Alzheimer Type Dementia (Topic) or Dementia, Alzheimer-Type (Topic) or Alzheimer's Diseases (Topic) or Alzheimer Diseases (Topic) or Alzheimers Diseases (Topic) or Alzheimer Dementia (Topic) or Alzheimer Dementias (Topic) or Dementia, Alzheimer (Topic) or Alzheimer's Disease (Topic) or Dementia, Senile (Topic) or Senile Dementia (Topic) or Dementia, Alzheimer Type (Topic) or Alzheimer Type Dementia (Topic) or Sclerosis, Alzheimer (Topic) or Dementia, Primary Senile Degenerative (Topic) or Dementia, Presenile (Topic) or Presenile Dementia (Topic) or Acute Confusional Senile Dementia (Topic) or Senile Dementia, Acute Confusional (Topic) or Alzheimer Disease, Early Onset (Topic) or Early Onset Alzheimer Disease (Topic) or Presenile Alzheimer Dementia (Topic) or Alzheimer Disease, Late Onset (Topic) or Late Onset Alzheimer Disease (Topic) or Alzheimer's Disease, Focal Onset (Topic) or Focal Onset Alzheimer's Disease (Topic) or Familial Alzheimer Disease (Topic) or Alzheimer Disease, Familial (Topic) or Familial Alzheimer Diseases (Topic) (331,329)

#2 TS=(Antioxidants OR Vitamins OR Vitamin E OR Ascorbic Acid OR Tocopherols OR Selenium OR Zinc OR Ubiquinone OR Acetylcysteine OR Carnitine OR Melatonin OR Glutathione OR Carotenoids OR Arginine OR Resveratrol OR AB=(Antioxidants OR Vitamin OR vitamin E OR tocopherol* OR alpha tocopherol* OR tocotrienol OR vitamin C OR ascorbic acid OR ascorb* OR selenium OR selen* OR zinc OR zinc* OR ubiquinone OR ubiquinol OR coenzyme Q10 OR CoQ10 OR Acetylcysteine OR Carnitine OR carnitene OR melatonin OR Glutathione OR GSH OR carotene OR betacarotene OR Arginine OR resveratrol* OR Evening Primrose Oil OR VitaminD OR curcumin) (1,864,947)

#3 ((((((((TS=(Randomized Controlled Trial OR Controlled Clinical Trial OR Clinical Trials as Topic)) OR AB=(randomized OR placebo OR randomly OR trial)) NOT TS=(animals)) NOT AB=(mice)) NOT AB=(mouse)) NOT AB=(fish)) NOT AB=(review)) NOT TS=(rats)) NOT TS=(rabbit) (1,824,014)

#4 #1 and #2 and #3 (962)

**Appendix 4. The Cochrane library search strategy**

**Searched 2025/8/21**

#1 (Alzheimer Disease):ti,ab,kw OR (Alzheimer Syndrome):ti,ab,kw OR (Alzheimer-Type Dementia):ti,ab,kw OR (Alzheimer Type Dementia):ti,ab,kw OR (Dementia, Alzheimer-Type):ti,ab,kw (Word variations have been searched) 14938

#2 (Alzheimer's Diseases):ti,ab,kw OR (Alzheimer Diseases):ti,ab,kw OR (Alzheimers Diseases):ti,ab,kw OR (Alzheimer Dementia):ti,ab,kw OR (Alzheimer Dementias):ti,ab,kw (Word variations have been searched) 15175

#3 (Dementia, Alzheimer):ti,ab,kw OR (Alzheimer's Disease):ti,ab,kw OR (Dementia, Senile):ti,ab,kw OR (Senile Dementia):ti,ab,kw OR (Dementia, Alzheimer Type):ti,ab,kw (Word variations have been searched) 15372

#4 (Alzheimer Type Dementia):ti,ab,kw OR (Sclerosis, Alzheimer):ti,ab,kw OR (Dementia, Primary Senile Degenerative):ti,ab,kw OR (Dementia, Presenile):ti,ab,kw OR (Presenile Dementia):ti,ab,kw (Word variations have been searched) 1517

#5 (Acute Confusional Senile Dementia):ti,ab,kw OR (Senile Dementia, Acute Confusional):ti,ab,kw OR (Alzheimer Disease, Early Onset):ti,ab,kw OR (Early Onset Alzheimer Disease):ti,ab,kw OR (Presenile Alzheimer Dementia):ti,ab,kw (Word variations have been searched) 253

#6 (Alzheimer Disease, Late Onset):ti,ab,kw OR (Late Onset Alzheimer Disease):ti,ab,kw OR (Alzheimer's Disease, Focal Onset):ti,ab,kw OR (Focal Onset Alzheimer's Disease):ti,ab,kw OR (Familial Alzheimer Disease):ti,ab,kw (Word variations have been searched) 1219

#7 #1 OR #2 OR #3 OR #4 OR #5 OR #6 15400

#8 (Selenium):ti,ab,kw OR (Zinc):ti,ab,kw OR (Ubiquinone):ti,ab,kw OR (Acetylcystein):ti,ab,kw OR (Carnitine):ti,ab,kw (Word variations have been searched) 14991

#9 (Melatonin):ti,ab,kw OR (Glutathione):ti,ab,kw OR (Carotenoids):ti,ab,kw OR (Arginine):ti,ab,kw OR (Resveratrol):ti,ab,kw (Word variations have been searched) 16902

#10 #8 OR #9 30635

#11 #10 AND #7 302
